# Zoogeography of South American Forest-Dwelling Bats: Disjunct Distributions or Sampling Deficiencies?

**DOI:** 10.1371/journal.pone.0133276

**Published:** 2015-07-17

**Authors:** Patrício Adriano da Rocha, Stephen Francis Ferrari, Anderson Feijó, Sidney Feitosa Gouveia

**Affiliations:** 1 Department of Ecology, Federal University of Sergipe–UFS, CEP 49100–000, São Cristóvão, Sergipe, Brazil; 2 Graduate Program in Zoology, Department of Systematics and Ecology, Federal University of Paraíba—UFPB, CEP 58059–900, João Pessoa, Paraíba, Brazil; University of Porto, PORTUGAL

## Abstract

Many forest-dwelling bats are purported to be widespread in South America, although records are scant from the vast diagonal belt of dry ecosystems that straddles the continent, implying possible sampling deficiencies. Here, we investigate this possibility in the case of four species of bat (*Centronycteris maximiliani*, *Lampronycteris brachyotis*, *Peropteryx kappleri* and *Trinycteris nicefori*), evaluating whether their disjunct present-day distributions reflect their true zoogeographic characteristics or the subsampling of intermediate zones. We use environmental niche modelling (ENM) in an ensemble approach, combining four different modeling techniques, and using niche descriptors based on climatic and remote sensing data, to estimate the potential distribution of the four species. The models indicate that all four species have disjunct distributions in the Amazon and Atlantic forest biomes. The one possible exception is *P*. *kappleri*, which the models indicated might potentially occur in humid forest enclaves in western Brazil and eastern Bolivia. The present-day distribution of the species may date back to the Plio-Pleistocene, when the forested biomes of South America were more extensive and connected. Further studies of different chiropteran lineages may provide additional insights into the historic processes of faunal interchange between the Amazon and Atlantic forest biomes.

## Introduction

Biogeographic patterns of continental biotas have been forged by cycles of environmental (mainly climatic) change, yielding complex patterns of species distributions in existing biomes [1,2]. These continuous transformations, coupled with the evolutionary history of the clades, have resulted in a mixture of species with both broad and narrow geographic ranges [3]. These individual patterns have received increasing attention from evolutionary biologists, especially in the context of phylogeographic studies [4]. However, the lack of reliable data on the occurrence of most clades precludes the conclusive definition of biogeographical patterns. Vast areas remain poorly sampled, providing inconclusive evidence on the history of colonization of certain species or their absence from specific regions. This distributional data deficiency has recently been referred to as Wallacean shortfall [5].

In case of South American biomes, for example, the humid formations (the Amazon and Atlantic rainforests) are separated by a diagonal band of more arid environments, the Caatinga, Chaco, and Cerrado [6]. There is substantial evidence from geological, palynological, botanical and zoological studies that the humid biomes expanded and retracted repeatedly during different epochs, coming into contact with one another on many occasions [7–12]. Vivo [13] suggested that mammals currently found in the Amazon and Atlantic forests were once common in the region now dominated by the Caatinga, being led to extinction by the establishment of the semiarid conditions that persist to the present day.

Some species of bats are able to disperse over long distances and many species are known to occur in both Amazon and Atlantic rainforests. One straightforward conclusion is that they constitute additional examples of species that migrated between these biomes during a prior period of connectivity and interchange, although Gardner [3] has proposed the existence of a corridor between Amazon and Atlantic forests northern running across northern Brazil. However, most of these small-bodied species appear to have low population densities, at least based on the available data from mist-netting studies. They also have not been recorded in the regions where they are believed to occur, in particular the limited zone of more humid conditions that ranges along the northern coast of Brazil between the two rainforest biomes.

Ecological niche modeling (ENM) and species distribution modeling (SDM) are widely-used and effective approaches to the analysis of ecological questions on species with uncertain geographic distributions. The suite of ENM techniques has become ubiquitous in modern ecology, with major applications in the fields of climate change, historical biogeography, and conservation planning [14–18]. The ENM methods now available range from simple, correlative approaches that aim to build bioclimatic envelopes of the spatial occurrence of species (e.g. [19, 20]) to more complex procedures based on artificial intelligence [21,22].

More recently, a novel strategy has been proposed, in which different techniques are combined to provide a consensus solution. This approach, known as “ensemble forecasting” [23], aims to circumvent the deficiencies and potential biases of each individual method, and has been conceived to improve the reliability of projections of future species distributions under climate shifts (e.g. [24–26]). The reasoning nevertheless applies as effectively to current conditions [27]. The essence of ensemble modeling is to average out predictions from different approaches as a means of reducing the uncertainties generated by varying initial conditions, model classes, parameters, and boundary definitions (see [23] for further details).

In view of the uncertainties on the present-day distribution of many chiropterans, we employ here an ensemble ENM protocol for the analysis of the data on four bat species that are posited to occur in South American cloud forest enclaves within the dry biomes that separate the principal rainforest biomes [28,29], *Centronycteris maximiliani* (Fischer, 1829), *Peropteryx kappleri* Peters, 1867, *Lampronycteris brachyotis* Dobson, 1879, *Trinycteris nicefori* Sanborn, 1949. The primary objective of this analysis is to assess whether the absence of these species from more arid environments represents their true, disjunct distribution or simply reflect the inadequate sampling of the intervening region.

## Material and Methods

**Fig 1:**
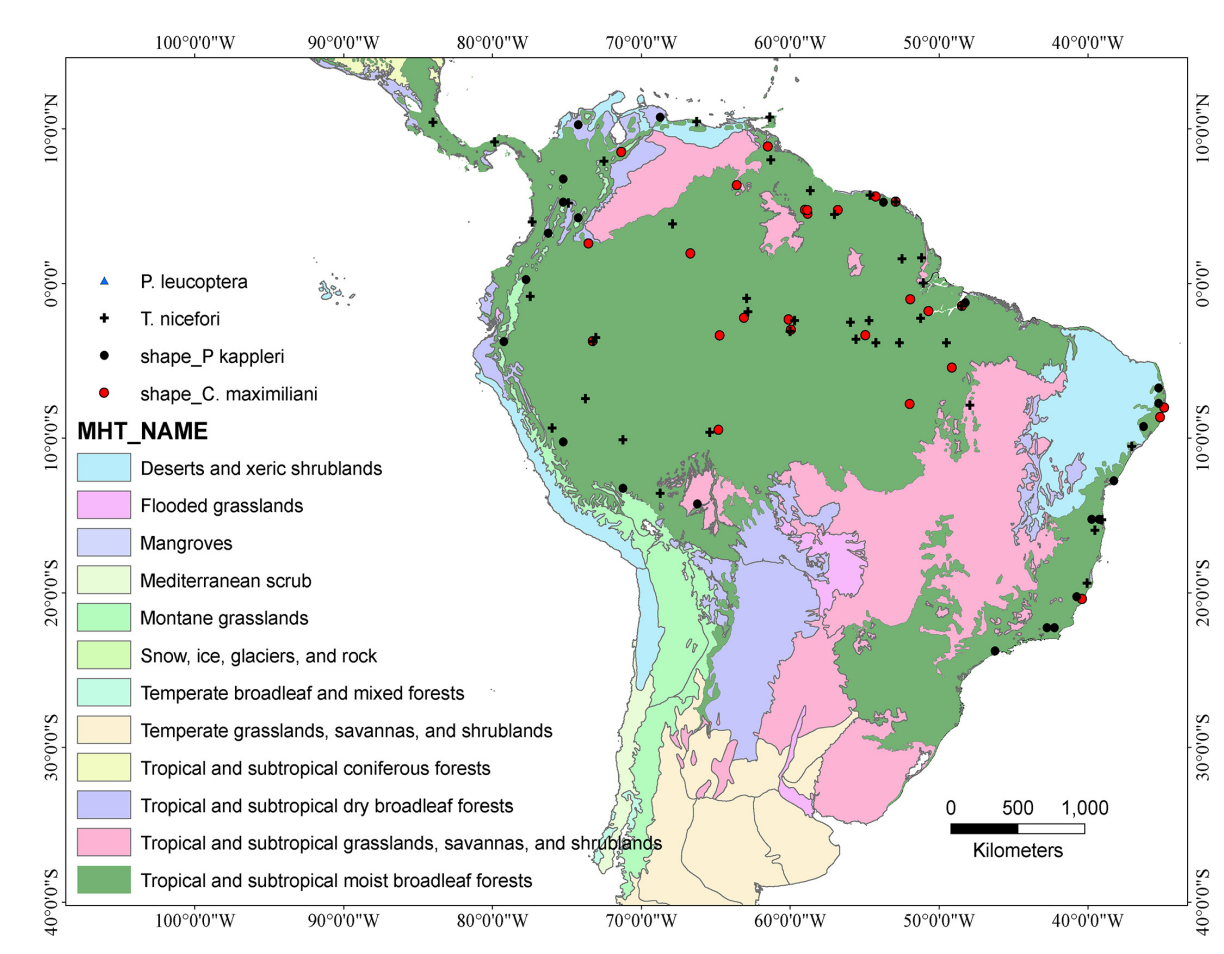
Distribution of South American biomes (according to the World Wide Fund for Nature) showing the point locality records of all four species used to build the ensemble niche models presented here.

We gathered occurrence data (geographic coordinates) of the four study species from published studies (S1, S2, S3 and S4 Tables), and we overlaid these locality point data onto a grid cell map of South America with 0.25° × 0.25° spatial resolution (Fig 1). The number of records for a given species varied from 23 to 34. As in other studies of species distribution modeling, we used a set of seven environmental variables that we believed to be the most relevant descriptors of the ecological niches of the four species, and consequently, of their spatial distribution. These included five bioclimatic variables (mean annual temperature, amplitude of diurnal temperatures, seasonality of annual temperatures, and precipitation of the driest and the wettest months). We also used two variables to describe the vegetation cover (as all the bat species inhabit forests), both based on the global model of Leaf Area Index (LAI: [30]). The LAI measures were arranged monthly for the year 2010. We estimated the mean annual LAI and the LAI of the driest month, as a proxy for the principal vegetation type and the difference between deciduous and evergreen forest, respectively. Bioclimatic variables were drawn from a high-resolution interpolated surface data series with a spatial resolution of approximately 20 km (1950–2000; see [31]). The raw LAI products are derived from the Moderate-Resolution Imaging Spectroradiometer (MODIS) satellite, which estimates the surface reflectance of the land surface at a 0.5 km spatial resolution with an 8-day sampling interval (see further details in [32]). We rescaled all these variables to the 0.25° cell grid prior to the analyses, to match the matrix of species occurrence data.

We used an ensemble approach to model the distribution of the study species, involving four methods: Bioclim [ 19 ], Mahalanobis distance [20], Genetic Algorithm for Rule Set Production, or GARP [33], and Maximum Entropy, Maxent [34]. Briefly, the Bioclim and Mahalanobis distances are based on the construction of bioclimatic envelopes based on a species’ occurrence records and the environmental conditions of these localities. The GARP and Maxent models are more sophisticated, being based on both occurrence and pseudo-absence data, also considering local environmental conditions (see related references for further details). In the present study, however, we generated pseudo-absence for all the models in order to evaluate their performance through the receiving operating curve, or ROC (see below). The models generated for each species were then combined into a final weighted consensus model, i.e. the ensemble. For each model, the entire dataset was randomly partitioned into two subsets, a 75% training fraction, and a 25% test fraction, a procedure known as cross-validation [35]. This routine was repeated 10 times for each method, i.e., a tenfold cross-validation. The training outputs provided the niche projection, whereas the test fraction provided an evaluation of the models’ performance.

We assessed the performance of the consensus models through true skill statistics (TSS), which is given by the sum of the models’ sensitivity and specificity minus 1. Sensitivity and specificity refer, respectively, to the proportion of the presence and absence records identified correctly. We also evaluated individual models through the area under the ROC curve (AUC), which is a plot of the true positives (*y*-axis) against unity minus the false positives (*x*-axis) of a given model. It is obtained by increasing cutoffs that convert continuous values of “local suitability” into a binary presence/absence variable. The area under this curve–AUC–provides an estimate of the model’s performance by measuring its ability to predict true occurrence (maximum sensitivity) while minimizing the error of predicting pseudo occurrence, or maximum specificity ([36, 37], but see [38, 39]). In this case, AUC values close to 1.0 denote high quality models, whereas values below 0.7 indicate poor models [40]. Here, we defined the threshold of each model with an automatic search using the ROC and a 0.5 consensus threshold for the final ensemble. We ran all modeling procedures and analyses in the Bioensembles software [41].

## Results

The final consensus models for all four species had high TSS values–from 0.926 for *T*. *nicefori* to 0.967 for *C*. *maximiliani*–indicating very good model fit in all cases. Individual models derived from the different approaches were also of high to very high quality, as indicated by the AUC values, ranging from 0.78 in the Mahalanobis distance model for *L*. *brachyotis* to 1.0 in the GARP models generated for all four species (Table 1).

**Table 1:** AUC values of the different models for each of the study species.

Models	BIOCLIM	Mahalanobis distance	GARP	Maxent
*C*. *maximiliani*	0.85	0.82	1	0.88
*P*. *kappleri*	0.88	0.81	1	0.95
*L*. *brachyotis*	0.91	0.78	1	0.92
*T*. *nicefori*	0.85	0.82	1	0.89

**Fig 2:**
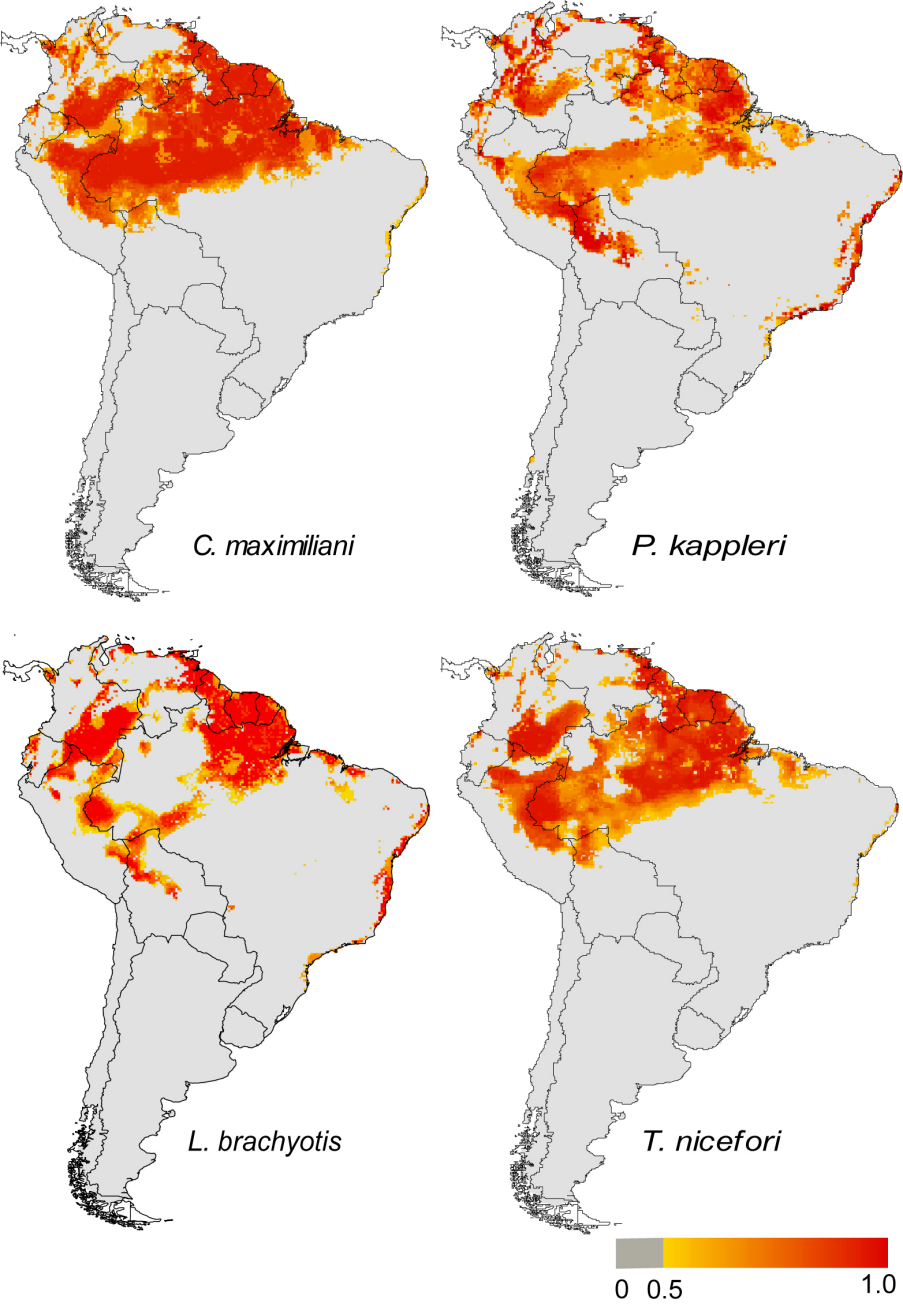
Final consensus models for the potential distribution of the four study species (labeled) in South America based on the consensus of four modeling techniques (Bioclim, Mahalanobis distances, GARP, and Maxent). All models portray the varying suitability values across space, from a lower 0.5 consensus threshold to unity.

The models described clear, disjunctive geographical distributions for two species–*C*. *maximiliani* and *T*. *nicefori*–including most of the Amazon and the northern extreme of the Atlantic forest. These predicted ranges varied only slightly in their total extension and overlap, with a marked congruence, in particular in the Amazon basin (Fig 2). In the two cases, the potential distribution of the species in the Atlantic forest was also greatly reduced, even considering their known distribution in this biome.

By contrast, the models indicated potential connections for the populations of *P*. *kappleri* and *L*. *brachyotis* (Fig 2). In the case of *P*. *kappleri*, there is a sparsely-distributed corridor of more humid enclaves stretching between southern Bolivia, eastern Paraguay, and southern Brazil. For *L*. *brachyotis*, the corridor extends through the humid enclaves of northeastern Brazil. The potential distribution of these species also extended considerably in the eastern and southern limits of the Atlantic forest biome, in comparison with their known occurrence in this region.

## Discussion

Our analyses have shown that three of the four chiropteran species analyzed indeed appear to have a disjunct distribution in the humid forests of South America. The one possible exception is *P*. *kappleri*, which may find suitable–but widely scattered–habitats between the southern limits of the Amazon and Atlantic forest domains. Overall, these results contradict the conclusion that the gap in the distributions of the species may be related to the subsampling of populations, and in particular the possible existence of a corridor of dispersion across northern Brazil [3]. Regarding the potential occurrence of *L*. *brachyotis* and *P*. *kapppleri* in corridors of scattered mesic forest enclaves bridging the northern and southern gaps, respectively, the models may reflect either later historical dispersal routes arising during periods of more humid climate or relict populations. For example, the enclaves identified within the dry diagonal as being suitable for *L*. *brachyotis* coincide with more mesic areas, such as the Chapada do Araripe and Baturite forests in Ceará, northeastern Brazil. The floristic characteristics of the latter are more closely linked to the Amazon and Atlantic forests than to those of other forest enclaves in the Caatinga [42].

In addition to reinforcing the classification of all four bat species as strict forest dwellers, the results of this study emphasize the importance of vicariant events in the determination of the present-day distribution of the species. In addition to Gardner’s [3] northern coastal corridor, there is considerable evidence of a southern route, as well as a central corridor during periods of milder climate [12, 43, 44]. Batalha-Filho et al. [12] identified two routes that enabled birds to interchange between the Amazon and Atlantic forests, one in the mid to late Miocene in the southern portion of the biomes, and a later route during the Pliocene–Pleistocene transition within the dry diagonal of the present day. Considering their comparable dispersal capabilities, birds and bats are likely to have congruent patterns of species intrusion between formations.

These conclusions may also have important implications for the taxonomic classification of these species. While they are clearly very closely related, the distinct populations appear to have been isolated from one another for long periods, raising potential doubts with regard to their species status, especially in light of studies of the recently-described species *Dryadonycteris capixaba* [45, 46]. This species was described from specimens collected from the Atlantic forest, identified previously as *Choeroniscus minor*. Subsequently, Rocha et al. [46] concluded that all the records of *C*. *minor* from the Atlantic forest actually refer to populations of *D*. *capixaba*, restricting the range of *C*. *minor* to the Amazon forest. The results of the present study suggest that other taxa, including those analyzed here, may be in a similar situation.

While ecological niche modeling has a range of applications in ecology, it was originally applied to the definition of the potential distribution of poorly-known species [14]. We have shown that this analytical tool may be equally useful for the differentiation of biogeographical processes from sampling deficiencies [47], reinforced by the highest degree of accuracy (i.e. TSS and AUC values) obtained by the ensemble approach. One important caveat here is that species niches may have been underestimated precisely because of the lack of records from intermediate (i.e. non-forested) conditions, which might have caused an environmental bias, and hence an incomplete niche characterization [48]. However, we do not believe that this is the case for the four species studied here, mainly because all the occurrence localities are restricted to rainforest habitats, and none at all coincide with adjacent savanna or scrub habitats, such as those found at the southern and northern edges of the Amazon biome (Fig 1). This also contradicts the idea of circular thinking affecting this study, i.e., biases in the data causing a bias toward a particular environmental setting in the model. Of course, models in general are subject to many sources of error, but we have attempted to avoid estimation biases as much as possible by adopting an ensemble framework. This strategy is especially appropriate for the avoidance of many potential sources of model bias, including both over- and under-prediction of the potential distribution of a species [23].

Indeed, forested habitat was a key factor in the characterization of the species’ niches. The annual mean LAI or the LAI of the driest month appeared among the better descriptors of species distributions, according to the estimates of relative contributions of the environmental variables provided by the Maxent models (Table 2). This highlights the importance of remote sensing data for the study of animal ecology in general [49], including niche models [50], and the relevance of forest remnants for these species, in particular. In fact, we have shown here that three of the four study species face reduced habitat suitability in the Atlantic forest (Fig 2), reflecting the relatively poor conservation status of this eastern Brazilian biome.

**Table 2:** Heuristic estimate of relative contribution of the environmental variables model for all four species as given by the Maxent output models.

Variables	*C*. *maximiliani*	*P*. *kappleri*	*L*. *brachyotis*	*T*. *nicefori*
Annual mean LAI	**51.5**	20	4.5	5.7
Driest month LAI	17.6	**29.6**	6.4	**39.4**
Mean temperature	0.1	5.2	0.1	0.8
Temp. seasonality	1.4	6.8	10.2	5.5
Temp. range	17.6	8.4	**78.1**	25.4
Precip. wettest month	0	0.4	0.3	1.7
Precip. driest month	2.5	**29.6**	0.1	21.5

In summary, we have shown that all four species of bat may indeed be confined to the forested environments of tropical South America and that their current distribution is not the product of sampling deficiencies, except possibly in one case. It seems likely that the present disjunct distribution of all four species is the product of the loss of a dispersal corridor that connected the Amazon and Atlantic forests no later than the Plio–Pleistocene. While the exact timing of this process and the dispersal routes of these clades have yet to be defined, the results of the present study provide important insights into the history of faunal interchange–specifically of chiropterans–between forested South American biomes.

## Supporting Information

S1 Table Recording localities for *Lampronycteris brachyotis* used in modeling analyses. (DOC)

S2 Table Recording localities for *Trinycteris nicefori* used in modeling analyses. (DOC)

S3 Table Recording localities for *Cetronycteris maximiliani* used in modeling analysis. (DOC)

S4 Table Recording localities for *Peropteryx kappleri* used in modeling analysis. (DOC)
