# Correction: Zoogeography of South American Forest-Dwelling Bats: Disjunct Distributions or Sampling Deficiencies?

**DOI:** 10.1371/journal.pone.0136808

**Published:** 2015-08-20

**Authors:** Patrício Adriano da Rocha, Stephen Francis Ferrari, Anderson Feijó, Sidney Feitosa Gouveia

There is an error in Fig 1. The legend within the figure is incorrect. Please view the correct Fig 1 here.

**Fig 1:**
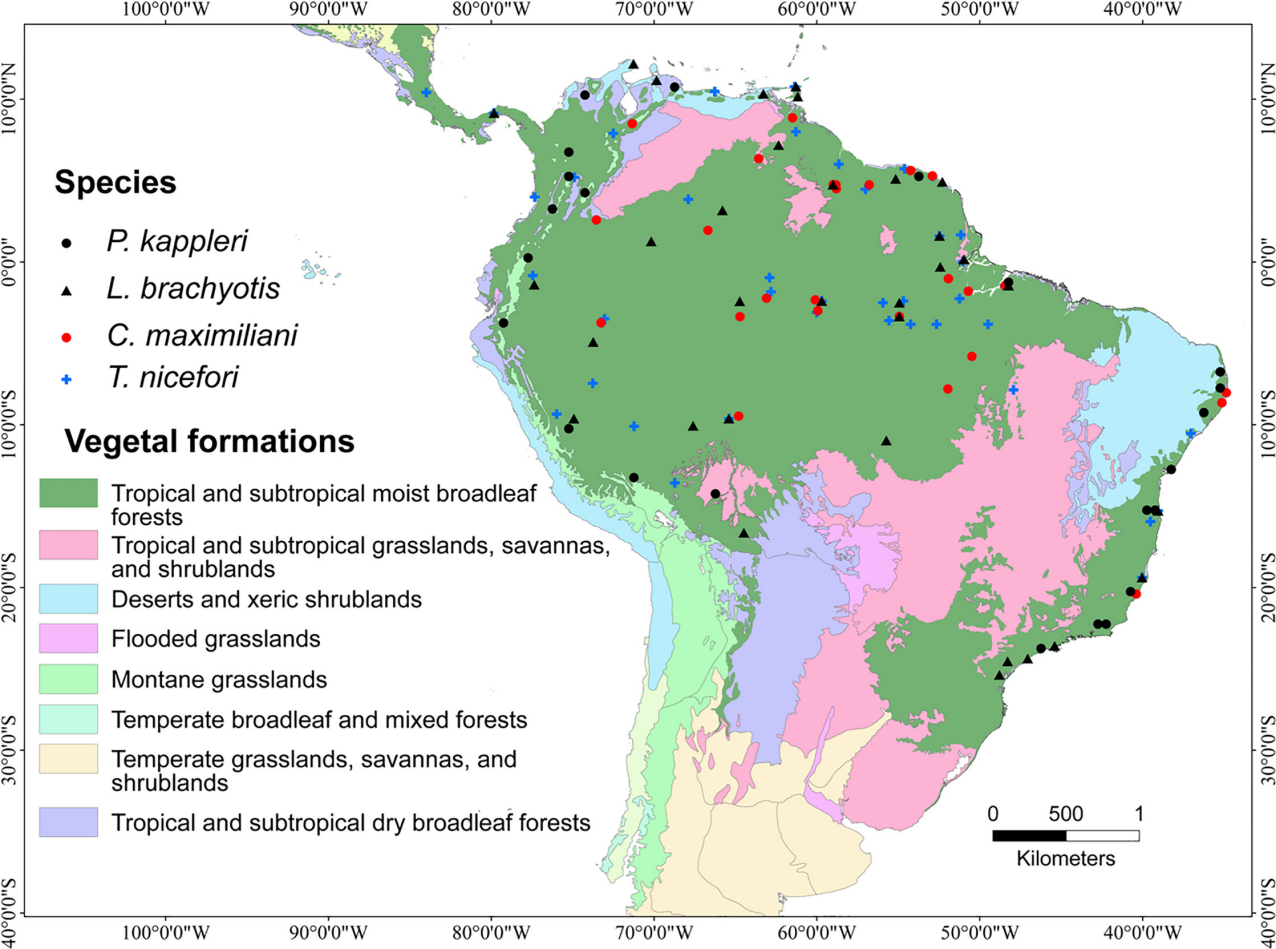
Distribution of South American biomes (according to the World Wide Fund for Nature) showing the point locality records of all four species used to build the ensemble niche models presented here.
