# Supplementary material for: Zoogeography of South American Forest-Dwelling Bats: Disjunct Distributions or Sampling Deficiencies?
Source: PLoS One. 2015 Jul 17;10(7):e0133276. doi: 10.1371/journal.pone.0133276 (PMC4505876; doi:10.1371/journal.pone.0133276)
Supplement: S1 Table — (DOC) [file pone.0133276.s001.doc]

S1Table. Recording localities for *Lampronycteris* *brachyotis* used in modeling analyses.

| **Reference** | **Latitude** | **Longitude** | **Locality** |
| --- | --- | --- | --- |
| 1 | 12.16666667 | -71.26666667 | Nazareth, Guajira, Colombia |
| 1 | 1.266666667 | -70.18333333 | Durania, Vaupés, Colombia |
| 2 | 11.16666667 | -69.85 | 19 km NW of Urama, Falcón, Venezuela |
| 2 | 7.183333333 | -62.36666667 | Los Patos, 25 km SE El Manteco, Bolívar,Venezuela |
| 2 | 3.166666667 | -65.81666667 | Tamatama, Rio Orinoco, Amazonas, Venezuela |
| 3 | 10.33333333 | -63.3 | Managas, 40km NW Carapito, Sucre, Venezuela |
| 4 | 10.78333333 | -61.3 | Blanchisseuse, Trinidad and Tobago |
| 5 | 10.16666667 | -61.18333333 | Victoria-Mayaro Forest Reserve, Trinidad and Tobago |
| 6 | 4.75 | -59.01666667 | Iwokrama Forest, Potaro-Siparuni, Pakatau falls, Guyana |
| 7 | 5.1 | -55.18333333 | Gros, Brokopondo, Suriname |
| 8 | 4.916666667 | -52.31666667 | Cayenne, French Guiana |
| 9 | -1.4 | -77.36666667 | Tarangaro,Pastaza Province, Ecuador |
| 10 | -4.916666667 | -73.75 | Loreto, Jenaro Herrera, Peru |
| 11 | -9.616666667 | -74.93333333 | Panguana Biological Station, Huanuco, Peru |
| 12 | -16.63333333 | -64.5 | Yapacaní, Provincia Ichilo, Bolivia |
| 13 | 1.6 | -52.48333333 | Parque Nacional Montanhas do Tumucumaque, Amapa, Brazil |
| 14 | 0.166666667 | -50.98333333 | APA Rio Curiaú - Macapá, Amapa, Brazil |
| 15 | -0.3 | -52.43333333 | Reserva de Desenvolvimento Sustentável do Rio Iratapuru, Amapa, Brazil |
| 16 | -1.45 | -48.25 | Área de Pesquisas Ecológicas do Guamá, Belém, Pará, Brazil |
| 17 | -2.5 | -54.95 | Alter do Chão, Pará, Brazil |
| 18 | -3.35 | -54.95 | Floresta Nacional dos Tapajós, Pará, Brazil |
| 19 | -2.4 | -59.71666667 | Manaus, Amazonas, Brazil |
| 20 | -2.416666667 | -64.75 | Reserva de Desenvolvimento Sustentável Amanã, Amazonas, Brazil |
| 21 | -10.06666667 | -67.61666667 | Senador Guiomard, Acre, Brazil |
| 22 | -10.96666667 | -55.75 | Nova Canaã do Norte, Mato Grosso, Brazil |
| 23 | -15.28333333 | -39.06666667 | Una, Bahia, Brazil |
| 24 | -19.38333333 | -40.05 | Linhares, Espirito Santo, Brazil |
| 25 | -24.53333333 | -48.3 | Pedro Cubas, Eldorado Paulista, São Paulo, Brazil |
| 25 | -23.58333333 | -45.41666667 | Parque Estadual Serra do Mar, São Paulo, Brazil |
| 28 | -24.36666667 | -47.06666667 | Estação Ecológica Juréia, Itatins,-Brazil |
| 28 | -25.36666667 | -48.8 | Reserva Natural Morro da Mina, Parana, Brazil |

**References**

1. Marinkelle CJ, Cadena A (1972) Notes on bats new to the fauna of Colombia. Mammalia 36:49–58.
2. Handley CO (1976) Mammals of the Smithsonian Venezuelan Project. Brigham Young Univ. Sci. Bull., biol. Ser. v. 20(5): 1–89.
3. Arnold ML, Baker RJ, Honeycutt RL (1983) Genic differentiation and Phylogenetic relationships within two New World bat genera. Biochem. Syst Ecol 11(3): 295–303.
4. Carter CH, Genoways HH, Loregnard RS, Baker RJ (1981) Observations on Bats from Trinidad, with a Checklist of Species Occurring on the Island. Occas pap Mus Nat Hist 179: 1–28.
5. Clarke FM, Pio DV, Racey PA (2005) A Comparison of Logging Systems and Bat Diversity in the Neotropics. Conserv Biol19(5): 1194–1204.
6. Lim BK, Engstrom MD (2001) Species diversity of bats (Mammalia: Chiroptera) in Iwokrama Forest, Guyana, and the Guianan subregion: implications for conservation. Biodivers Conserv. 10: 613–657.
7. Husson AM (1978) The mammals of Suriname.Zoölogische Monographieën van het Rijksmuseum van Natuurlijke Historie No. 2. Leiden: E. J. Brill, 160 p.
8. Brongniart A (1792) Catalogue de mammiferes envoyes de Cayenne par M. le Blond. Actes Soc Nat Hist Paris 1:115.
9. Tirira DG, Boada CE, Burneo SF (2010) Mammalia, Chiroptera, Phyllostomidae, *Lampronycteris* *brachyotis* (Dobson, 1879): First confirmed record for Ecuador. Check List 2(6): 237–238.
10. Solari S, Pacheco V, Vivar E (1999) Nuevos Registros Distribucionales de Murciélagos Peruanos. Rev Peru Biol 6(2):152–159.
11. Hutterer R, Verhaagah M, Diller J, Podloucky R (1995) An inventory of mammals observed at Panguana Biological Station, Amazonian Peru. Ecotropica 1:3–20.
12. AcostaSL, Aguanta AF (2005) Nota sobre um nuevo registro de murciélago (Lampronycteris brachyptis) para Bolívia. Kempffiana. Revista de divulgación cientifica em Historia Natural, Ecologia, Biogeografia & Taxonomia 1(1): 65–69.
13. Martins ACM, Bernard E (2008) Inventários biológicos rápidos da fauna de morcegos de cinco localidades do Parque Nacional Montanhas do Tumucumaque, Amapá. In: Bernard E, editor. Inventários Biológicos Rápidos no Parque Nacional Montanhas do Tumucumaque, Amapá, Brasil. RAP Bulletin of Biological Assessment 48. Conservation International, Arlington, VA. pp. 51–65.
14. Castro IJ (2009) Assembleia de morcegos (Mammalia: Chiroptera) da Área de Proteção Ambiental do Rio Curiaú, Amapá. Master Dissertation. Universidade Federal do Amapá, Amapá.
15. Martins ACM, Bernard E, Gregorin R, Silva WAS (2011) Filling data gaps on the diversity and distribution of Amazonian bats (Chiroptera): The case of Amapá, easternmost Brazil. Zoologia 28(2): 177–185.
16. Handley CO (1967) Bats of the canopy of an Amazonian forest. Atas do Simpósio sôbre a Biota Amazônica 5: 211–215.
17. Bernard E, Fenton MB (2002) Species diversity of bats (Chiroptera: Mammalia) in forest fragments, primary forests and savannas in Central Amazonia, Brazil. Can J Zool 80:1124–1140.
18. Castro-Arellano I, Presley SJ, Saldanha LN, Willig MR, Wunderle Jr JM (2007) Effects of reduced impact logging on bat biodiversity in terra firme forest of lowland Amazonia. Biol Conserv 138: 269–285.
19. Sampaio EM, Kalko EK, Bernard E, Rodríguez-Herrera B, Handley Jr CO (2003) A Biodiversity Assessment of Bats (Chiroptera) in a Tropical Lowland Rainforest of Central Amazonia, Including Methodological and Conservation Considerations. Stud Neotrop Fauna Environ 38(1): 17–31.
20. Pereira MJR, Marques JT, Palmerim JM (2010) Vertical stratification of bat assemblages in flooded and unflooded Amazonian forests. Curr Zool 56(4): 469–478.
21. Marciente R, Calouro AM (2009) Mammalia, Chiroptera, Phyllostomidae, Lampronycteris brachyotis (Dobson, 1879): First record in Acre, Brazil. Check List 5(4): 886–889.
22. Miranda JMD, Zago L, Carvalho F, Rubio MBG, Bernardi IP (2015) Morcegos (Mammalia: Chiroptera) da região do Médio Rio Teles Pires, Sul da Amazônia, Brasil. Acta Amaz 45(1) 2015: 89–100.
23. Faria D, Soares-Santos B, Sampaio E (2006) Bats from the Atlantic rainforest of Southern Bahia, Brazil. Biot Neotrop 6(2): 2–13.
24. Peracchi AL, Albuquerque ST (1993) Quirópteros do município de Linhares, estado do Espírito Santo, Brasil (Mammalia, Chiroptera). Rev Bras Biol 53(4): 575–581.
25. Taddei VA, Pedro WA (1996) Micronycteris brachyotis (Chiroptera, Phyllostomidae) from the state of São Paulo, Brazil. Rev Bras Biol 56(2): 217–222.
26. Geraldes MP (2005) Diversidade e estratificação altitudinal de conjuntos taxonômicos de morcegos na Mata Atlântica da Serra do Mar, São Paulo. PhD Thesis. Universidade de São Paulo, São Paulo.
27. Gimenez EA, Ferrarezzi H (2004) Diversidade de morcegos no sudeste da Mata Atlântica. In: Marques OAV, Duleba W, editors. Estação Ecológica Juréia-Itatins: ambiente físico, flora e fauna. 1a ed. Ribeirão Preto: Editora Holos, pp.314–330.
28. Scultori C, Dias D, Peracchi AL (2009) Mammalia, Chiroptera, Phyllostomidae, Lampronycteris brachyotis (Dobson, 1879): First record in the state of Paraná, southern Brazil. Check List. 5(4): 872–875.
