# Supplementary material for: Zoogeography of South American Forest-Dwelling Bats: Disjunct Distributions or Sampling Deficiencies?
Source: PLoS One. 2015 Jul 17;10(7):e0133276. doi: 10.1371/journal.pone.0133276 (PMC4505876; doi:10.1371/journal.pone.0133276)
Supplement: S2 Table — (DOC) [file pone.0133276.s002.doc]

S2Table. Recording localities for *Trinycteris nicefori* used in modeling analyses.

| **Reference** | **Latitude** | **Longitude** | **Locality** |
| --- | --- | --- | --- |
| 1 | 10.46666667 | -66.28333333 | Trujillo 19 kmN of Valera, Miranda, Birongo, Venezuela |
| 2 | 8 | -61.3 | Bolívar-Unidad V, Reserva Forestal Imataca, Venezuela |
| 3 | 13.05 | -67.91666667 | Inírida, Colombia |
| 4 | 7.9 | -72.51666667 | Norte de Santander, Cucuta, Colombia |
| 5 | 5.2 | -74.9 | Tolima, Mariquita, Colombia |
| 6 | 3.983333333 | -77.33333333 | Valle del Cauca, Bahía Málaga Naval Base, Colombia |
| 7 | 10.76666667 | -61.38333333 | Trinidad, Las Cuevas, Trinidad and Tobago |
| 8 | 6 | -58.66666667 | Cuyuni-Mazaruni, Bartica, 24 miles along Portaro Road, Guyana |
| 9 | 5.283333333 | -52.91666667 | Paracou, French Guiana |
| 10 | 5.716666667 | -54.63333333 | Marowijne, Moengo, 10 kilometers N and 24 km W, Suriname |
| 11 | 4.466666667 | -57.03333333 | Bakhuis, Suriname |
| 12 | -0.833333333 | -77.46666667 | Orellana -Santa Rosa de Arapino, Ecuador |
| 13 | -10.1 | -71.23333333 | Ucayali,Balta, Río Curanja, Peru |
| 14 | -9.35 | -75.98333333 | Huanuco, Tingo María, Hotel Turistas, Peru |
| 15 | -3.733333333 | -73.23333333 | Iquitos, Peru |
| 16 | -3.483333333 | -73.05 | Loreto,Puerto Indiana , Peru |
| 17 | -13.56666667 | -68.73333333 | La Paz, Bolivia |
| 18 | 1.6 | -52.48333333 | Parque Nacional Montanhas do Tumucumaque, Amapá, Brazil |
| 18 | 1.666666667 | -51.16666667 | Floresta Nacional do Amapá, Amapá, Brazil |
| 19 | 0.033333333 | -51.05 | Macapá, Amapá, Brazil |
| 20 | -1.45 | -48.5 | Belém, Pará, Brazil |
| 21 | -3.833333333 | -49.5 | Tucuruí, Pará, Brazil |
| 22 | -2.25 | -51.25 | FLONA Caxiuanã, Pará, Brazil |
| 13 | -3.833333333 | -52.66666667 | Altamira, Pará, Brazil |
| 23 | -3.833333333 | -54.25 | Parque Nacional da Amazônia, Pará, Brazil |
| 24 | -2.4 | -54.7 | Santarém, Pará, Brazil |
| 25 | -2.5 | -55.95 | Alter do Chão, Pará, Brazil |
| 26 | -3.6 | -54.98333333 | Parque Nacional do Tapajós, Amazonas, Brazil |
| 27 | -2.4 | -59.71666667 | Manaus, PDBFF, Amazonas |
| 28 | -3.1 | -60.01666667 | Manaus, Reserva Ducke, Amazonas, Brazil |
| 29 | -1.833333333 | -62.83333333 | Parque Nacional Jaú, Amazonas, Brazil |
| 30 | -0.966666667 | -62.91666667 | Barcelos, Amazonas, Brazil |
| 31 | -7.45 | -73.76666667 | Parque National da Serra do Divisor, Acre, Brazil |
| 32 | -9.616666667 | -65.41666667 | Porto Velho, Abunã, Rondonia, Brazil |
| 33 | -7.866666667 | -47.93333333 | Palmeirante, Tocantins, Brazil |
| 34 | -10.53333333 | -37.05 | RVS Mata do Junco, Sergipe, Brazil |
| 35 | -15.95 | -39.53333333 | Itapebi, Bahia, Brazil |
| 35 | -15.25 | -39.06666667 | Una, Bahia, Brazil |
| 36 | -19.41666667 | -40.05 | Linhares, Espírito Santo, Brazil |

**References**

1 Williams SL, Genoways HH (2008) Subfamily Phyllostominae Gray, 1825. In: Gardner AL, editor. Mammals of south America, volume I: marsupials, xenarthrans, shrews and bats. Chicago. The Univeristy of Chicago Press,pp. 255–300.

2 Ochoa GJ (1995) Los mamíferos de la Región de Imataca, Venezuela. Acta Cient Venez 46: 274–287.

3 Ferrer-Peréz A, Gutiérrez MB, Lasso CA (2009) Mamíferos de la Estrella Fluvial de Inírida: ríos Inírida, Guaviare, Atabapo y Orinoco (Colombia). Biota Colombiana 10(1-2): 209–218.

4 Sanborn CC (1949) Bats of the genus Micronycteris and its subgenera. Fieldiana Zool 31: 215–33.

5 Muñoz J (2001) Los murciélagos de Colombia. Systemática, distribución, descripción, historia natural y ecología. Medellín, Colombia: Editorial Universidad de Antioquia, 391 pp

6 Alberico MS (1987) Notes on distribution of some bats from southwestern Colombia. In: Patterson BD, Timm RM, editos. Studies in Neotropical mammalogy, essays in honor of Philip Hershkovitz, Fieldiana Zool., 39: 133–36

7 Carter CH, Genoways HH, Loregnard RS, Baker RJ (1981) Observations on bats from Trinidad, with a checklist of species occurring on the island. Occas Pap Tex Tech Univ Mus 72: 1–27

8 Hill JE (1965) Notes on bats from British Guiana, with the description of a new genus and species of Phyllostomidae. Mammalia 28(4): 553–572

9 Simmons NB, Voss RS (1998) The mammals of Paracou, French Guiana: A Neotropical lowland rainforest fauna, Part 1. Bull Am Mus Nat Hist 237: 1–219

10 Genoways HH, Williams SL (1979) Records of bats (Mammalia: Chiroptera) from Suriname. Ann Carnegie Mus 48: 323–335.

11 Lim BK (2009) Environmental Assessment at the Bakhuis Bauxite Concession: Small-Sized Mammal Diversity and Abundance in the Lowland Humid Forests of Suriname. Open Biology 2: 42–53

12 Albuja VL (1999) Murciélagos del Ecuador. 2nd ed. Quito: Cicetrónica Compañia Limitada Offset, 288 p

13 Voss RS, Emmons LH (1996) Mammalian diversity in Neotropical lowland rainforests: a preliminary assessment. Bull Am Mus Nat Hist 230: 1–115

14 Bowles JB, Cope JB, Cope EA (1979) Biological studies of selected Peruvian bats of Tingo Maria, Departmento de Huánuco. Trans Kans Acad Sci 82:1–10.

15 Klingbeil BT, Willig MR (2010) Seasonal differences in population-, ensemble- and community-level responses of bats to landscape structure in Amazonia. Oikos 119: 1654–1664

16 Pirlot P (1968) Chiropteres du Perou, specialement de haute-Amazonie. Mammalia 32(1): 86–96.

17 Anderson S (1997) Mammals of Bolivia: Taxonomy and distribution. Bull Am Mus Nat Hist 231: 1–652

18 Martins ACM, Bernard E, Gregorin R (2006) Inventários biológicos rápidos de morcegos (Mammalia, Chiroptera) em três unidades de conservação do Amapá, Brasil. Rev Bras Zool 23(4): 1175–1184.

19 Peracchi AL, Raimundo SDL, Tannure AM (1984) Quirópteros do território Federal do Amapá, Brasil (Mammalia: Chiroptera). Arquivos da Universidade Federal Rural do Rio de Janeiro 7(2): 89–100.

20 Bernard E, Tavares VC, Sampaio E. 2011. Updated compilation of bat species (Chiroptera) for the Brazilian Amazonia. Biota Neotrop 11: 35-46

21 Pine RH, LaVal RK, Carter DC, Mok WY (1996) Notes on the graybeard bat, *Micronycteris daviesi* (Hill) (Mammalia: Chiroptera: Phyllostomidae), with the first records from Ecuador and Brazil. In: Genoways HH, Baker RJ, editors. Contributions in Mammalogy: A Memorial Volume Honoring Dr. J. Knox Jones, Jr. Lubbock: Museum of Texas Tech University, pp. 183–190.

22 Marques-Aguiar SA, Aguiar GFS (2002) Interações de quirópteros em ecossistemas tropicais: perspectivas de estudo para Caxiuanã. In: Lisboa PLB,editor. Caxiuanã: populações tradicionais, meio físico e diversidade biológica. Belém: Museu Paraense Emílio Goeldi, pp.651–668.

23 Marques SA (1985) Novos registros de morcegos do Parque Nacional da Amazônia (Tapajós), com observações do período de atividade noturna e reprodução. Boletim do Museu Paraense Emílio Goeldi, série zoo 2: 71–83.

24 Bernard E (2001) Species list of bats (Mammalia: Chiroptera) of Santarém area, Pará State, Brazil. Rev Bras Zool 18(2): 455–463

25 Bernard E, Fenton MB (2002) Species diversity of bats (Chiroptera: Mammalia) in forest fragments, primary forests and savannas in Central Amazonia, Brazil. Can J Zool 80: 1124–1140

26 Presley SJ, Willig MR, Wunderle Jr M, Saldanha LN (2008) Effects of reduced-impact logging and forest physiognomy on bat populations of lowland Amazonian forest. J Appl Ecol 45(1): 14–25.

27 Sampaio EM, Kalko EKV, Bernard E, Rodríguez-Herrera B, Handley CO (2003) A biodiversity assessment of bats (Chiroptera) in a tropical lowland rainforest of Central Amazonia, including methodological and conservation considerations. Stud Neotrop Fauna E 38: 17–31

28 Reis NR, Peracchi AL (1987) Quirópteros da região de Manaus, Amazonas, Brasil (Mammalia, Chiroptera). Boletim do Museu Paraense Emílio Goeldi, Série Zoologia 3: 161–82.

29 Barnett AA, Sampaio EM, Kalko EKV, Shapley RL, Fischer E, Camargo G, Rodriguez-Herrera B (2006) Bats of Jaú National Park, central Amazônia, Brazil. Acta Chiropt 8(1): 103–128

30 Moratelli R, Dias D, Bonvicino CR (2010) Estrutura e análise zoogeográfica de uma taxocenose de morcegos no norte do estado do Amazonas, Brasil. Chiropt Neotrop 16(1): 661–671

31 Nogueira MR, Pol A, Peracchi AL (1999) New records of bats from Brazil with a list of additional species for the chiropteran fauna of the state of Acre, western Amazon basin. Mammalia 63(3): 363–368

32 Rocha PA, Garbino GST, Aires CC (2013) Update on the distribution of Trinycteris nicefori Sanborn, 1949 (Chiroptera: Phyllostomidae): new record for the Amazonia of Brazil. Check List 9(4): 785–789.

33 Nunes A, Marques-Aguiar SA, Saldanha N, Silva RS, Bezerra A (2005) New records on the geographic distribution of bat species in the Brazilian Amazonia. Mammalia 69: 109–115.

34 Brito DV, Bocchiglieri A (2012) Comunidade de morcegos (Mammalia, Chiroptera) no Refúgio de Vida Silvestre Mata do Junco, Sergipe, nordeste do Brasil. Biota Neotrop 12 (3):1-9.

35 Faria D, Soares-Santos B, Sampaio E (2006) Bats from the Atlantic rainforest of Southern Bahia, Brazil. Biot Neotrop 6(2): 2–13

36 Peracchi AL, Albuquerque ST (1993) Quirópteros do município de Linhares, Estado do Espírito Santo, Brasil (Mammalia, Chiroptera). Rev Bras Biol 53: 575–581
