# Supplementary material for: Zoogeography of South American Forest-Dwelling Bats: Disjunct Distributions or Sampling Deficiencies?
Source: PLoS One. 2015 Jul 17;10(7):e0133276. doi: 10.1371/journal.pone.0133276 (PMC4505876; doi:10.1371/journal.pone.0133276)
Supplement: S3 Table — (DOC) [file pone.0133276.s003.doc]

S3Table. Recording localities for *Cetronycteris maximiliani* used in modeling analysis.

| **Reference** | **Latitude** | **Longitude** | **Locality** |
| --- | --- | --- | --- |
| 7 | 5.283333 | -52.9167 | Paracou Field Station, French Guiana |
| 2 | 5.616667 | -54.25 | Marowijine, Suriname |
| 3 | 4.733333 | -56.8 | Bakhuis, Suriname |
| 4 | 4.5 | -58.8167 | Iwokrama Forest, Potaro-Siparuni, Guyana |
| 5 | 4.733333 | -58.85 | Clearwater Camp, Potaro-Siparuni, Guyana |
| 5 | 4.75 | -59.0167 | Pakatau Falls, Potaro-Siparuni, Guyana |
| 6 | 8.85 | -61.5 | Delta Amacuro, Venezuela |
| 6 | 6.35 | -63.5833 | Bolívar, Venezuela |
| 7 | 1.933333 | -66.7 | Amazonas, Buena Vista, Venezuela |
| 4 | 8.5 | -71.35 | San Juan, Mérida, Venezuela |
| 8 | 2.583333 | -73.55 | Serranía de La Macarena, Meta, Colombia |
| 9 | -3.73333 | -73.2667 | Iquitos, Loreto, Peru |
| 7 | -3.36667 | -64.7333 | Tefé, Amazonas, , Brazil |
| 10 | -2.23333 | -63.1 | Jaú National Park, Amazonas, Brazil |
| 7 | -2.33333 | -60.1167 | Biological Dynamics of Forest Fragments Project, Amazonas, Brazil |
| 11 | -3 | -59.95 | Manaus, Amazona, Brazil |
| 12 | -1.03333 | -51.9333 | Floresta Nacional do Amapá, Amapá , Brazil |
| 7 | -1.45 | -48.4667 | Pará, Belém, Brazil |
| 13 | -1.8 | -50.7167 | Estação Científica Ferreira Penna, Melgaço, Pará, Brazil |
| 14 | -3.35 | -54.95 | Floresta nacional Tapajos, Pará, Brazil |
| 15 | -7.8 | -51.9667 | Kayapó Center for Ecological Research (Pinkaití), Pará, Brazil |
| 10 | -5.8 | -50.5 | Marabá, Pará, Brazil |
| 7 | -8.03333 | -34.8667 | Recife, Pernambuco, Brazil |
| 1 | -8.65 | -35.15 | Rio Formoso, Pernambuco, Brazil |
| 7 | -20.4 | -40.4 | Rio Jucu, Espirito Santo, Brazil |

**Reference**

1 Feijó A (2010) A quiropterofauna dos estados da Paraíba e Pernambuco. Graduate Thesis. Universidade Federal da Paraiba, p 214.

2 Williams SL, Genoways HH, Groen JA (1983) Results of the Alcoa Foundation-Suriname Expeditions. VII. Records of mammals from central and southern Suriname. Ann. Carnegie Mus 52: 329–36.

3 Lim BK (2009) Environmental Assessment at the Bakhuis Bauxite Concession: Small Sized Mammal Diversity and Abundance in the Lowland Humid Forests of Suriname. Open Biology 2, 42–53.

4 Lim BK, Engstrom MD (2001) Species diversity of bats (Mammalia: Chiroptera) in Iwokrama Forest, Guyana, and the Guianan subregion: implications for conservation. Biodivers Conserv 10: 613–657.

5 Lim BK, Engstrom MD, Timm RM, Anderson RP, Watson LC (1999) First records of 10 bat species in Guyana and comments on diversity of bats in Iwokrama Forest. Acta Chiropterol 1:179–190.

6 Lim B, Tavares VC (2012) Species Richness, Biogeography, and Conservation Status Of Bats from the Guiana Subregion of Northern South America. Ecotropica 18: 105–118.

7 Simmons NB, Handley Jr CO (1998) A revision of *Centronycteris* Gray (Chiroptera: Emballonuridae) with notes on natural history. Am Mus Novit 3239: 1–28.

8 Cuervo-Díaz A, Hernández-Camacho J, Cadena GA (1986) Lista actualizada de los mamíferos de Colombia: anotaciones sobre su distribución. Caldasia15:471–502.

9 Hice CL, Solari S (2002) First record of *Centronycteris* *maximiliani* (Fisher, 1829) and two additional records of *C*. *centralis* Thomas, 1912 from Peru. Acta Chiropterol 4:217–20.

10 Barnett AA, Sampaio EM, Kalko EKV, Shapley RL, Camargo G, Rodriguez Herrera B (2006) Bats of Jaú National Park, central Amazônia, Brazil. Acta Chiropterol 8(1):103–128.

11 Reis NR (1984) Estrutura de comunidade de morcegos na região de Manaus, Amazonas. Rev Bras Biol 44: 247–54.

12 Martins ACM, Bernard E, Gregorin R (2006) Inventários biológicos rápidos de morcegos (Mammalia, Chiroptera) em três unidades de conservação do Amapá, Brasil. Rev Bras Zool 23: 1175–1184.

13 Marques-Aguiar SA, Aguila MV, Aguiar GFS, Saldanha N, Silva-Junior JS, Rocha MMB (2003) Caracterização e perspectivas de estudo dos quirópteros da Estação Científica Ferreira Penna- município de Melgaço- PA. Idéias e Debates 6:1–3.

14 Castro-Arellano I, Presleya SJ, Saldanha LN, Willig MR, Wunderle JM (2007) Effects of reduced-impact logging on bat biodiversity in terra firme forest of lowland Amazonia. Biol Cons 138, 269–285.

15 Peters SL, Malcolm JR, Zimmerman BL (2006) Effects of Selective Logging on Bat Communities in the Southeastern Amazon. Conserv Biol 20(5): 1410–1421.
