# Supplementary material for: Zoogeography of South American Forest-Dwelling Bats: Disjunct Distributions or Sampling Deficiencies?
Source: PLoS One. 2015 Jul 17;10(7):e0133276. doi: 10.1371/journal.pone.0133276 (PMC4505876; doi:10.1371/journal.pone.0133276)
Supplement: S4 Table — (DOC) [file pone.0133276.s004.doc]

S4Table. Recording localities for *Peropteryx* *kappleri* used in modeling analysis.

| **Reference** | **Latitude** | **Longitude** | **Locality** |
| --- | --- | --- | --- |
| 1 | -7.11532 | -34.861051 | João Pessoa, Paraiba, Brazil |
| 2 | -9.323372 | -36.474403 | Pedra Talhada, Alagoas, Brazil |
| 3 | -12.970382 | -38.512382 | Salvador, Bahia, Brazil |
| 4 | -15.595758 | -39.85758 | Poliraguá, Bahia, Brazil |
| 4 | -15.575101 | -39.287074 | Mascote, Bahia, Brazil |
| 5 | -22.620229 | -42.356919 | Reserva Biologica Poço das Antas, Rio de Janeiro, Brazil |
| 6 | -22.412324 | -42.966432 | Teresópolis, Rio de Janeiro, Brazil |
| 7 | -23.776508 | -45.512734 | São Sebastiao, São Paulo, Brazil |
| 8 | -8.132639 | -35.133626 | Mata do Camocim, Pernambuco, Brazil |
| 9 | -20.606167 | -41.194975 | Castelo, Espirito Santo, Brazil |
| 10 | -1.45502 | -48.502368 | Belém, Pará, Brazil |
| 11 | -14.2 | -66.616667 | Beni, Bolivia |
| 8 | -13.525384 | -71.945321 | Quispicanchi, Cusco, Peru |
| 12 | -13.65 | -71.08333333 | Pagoreni, Cusco, Peru |
| 13 | -10.590278 | -75.398611 | Oxampampa, Peru |
| 7 | -3.72 | -79.62 | Portovelo, El Oro, Ecuador |
| 14 | 0.662553 | -78.019539 | Puente piedra, Carchi, Ecuador |
| 15 | 4.628765 | -74.466423 | La mesa, Cundinamarca, Colombia |
| 16 | 7.198606 | -75.341218 | Guapa, Antioquio, Colombia |
| 17 | 10.411402 | -74.405661 | Magdalena, Colombia |
| 18 | 3.420556 | -765222222 | Cali, Valle del Cauca, Colombia |
| 19 | 5.250016 | -75.50003 | Finca los naranjos, Caldas, Colombia |
| 20 | 10.90649 | -68.762039 | Riecito, Falcon, Venezuela |
| 21 | 5.505068 | -54.079345 | Albina, Marowijne, Suriname |

**Reference**

1. Feijó JA, Langguth A (2011) Lista de Quirópteros da Paraiba, Brasil com 25 novos registros. Chiropt Neotrop 17(2): 1055–1062.

2 Sousa MAN, Langguth A, Gimenez EA (2004) Mamí́feros dos brejos de altitude Paraíba e Pernambuco. In: Porto KC, Cabral JJP, Tabarelli M,editors. Brejos de altitude em Pernambuco e Paraíba. Ministério do Meio Ambiente, Brasília, pp 229–254.

3 Vieira CO (1955) Lista remissiva dos mamíferos do Brasil. Arq. Zool. Estado São Paulo 8: 341–474.

4 Faria D, Soares-Santos B, Sampaio E (2006) Bats from the Atlantic rainforest of Southern Bahia, Brazil. Biot Neotrop 6(2): 2–13.

5. Brito D, Oliveira LC, Mello MA (2004) An overview of mammalian conservation at Poço das Antas Biological Reserv, southeastern Brazil. J Nat Conserv 12: 219-228.

6 Vieira CO (1942) Ensaio monográfico sobre os quirópteros do Brasil. Arq. Zool. Estado São Paulo 3: 219–471.

7 Sanborn CC (1937) American bats of the subfamily Emballonurinae. Field Mus. Nat. Hist., zool. ser., 20:321–354

8. Hood C, Gardner AL (2008) Family Emballonuridae Gervais, 1856. In: Gardner AL, editor. Mammals of south America, volume I: marsupials, xenarthrans, shrews and bats. Chicago. The Univeristy of Chicago Press, pp. 188–207.

9 Ruschi A (1951) Morcegos do estado do Espírito Santo. Família Emballonuridae, chave analítica para os gêneros, espécies, e subspecies representados no estado do Espírito Santo. Descricão de *Peropteryx macrotis macrotis* e *Peropteryx kappleri*. Bol. Mus. Biol. Prof. Mello Leitão, zool., no. 8:1–13.

10 Mok WY, Wilson DE, Lacey LA, Luizão RCC (1982) Lista atualizada de quirópteros da Amazônia brasileira. Acta Amaz. 12:817-23.

11 Anderson S (1997) Mammals of Bolivia, taxonomy and distribution. Bull. Amer. Mus. Nat. Hist.231:1–652

12 Solari S, Vivar E, Velazco PM, Rodríguez JJ, Wilson DE, Baker RJ, Mena JL (2001) The small mammal community of the Lower Urubamba Region, Peru. In: Alonso A, Dallmeier F, Campbell P, editors. Urubamba: The biodiversity of a Peruvian rainforest. Washington, DC. The Smithsonian Institution, pp: 171–181.

13 Tuttle (MD) 1970. Distribution and zoogeography of Peruvian bats, with comments on natural history. Univ. Kansas Sci. Bull. 49:45–86.

14 Albuja VL (1999) Murciélagos del Ecuador. 2nd ed. Quito,Ecuador: Cicetrónica Cía. Ltda. Offset, 288 pp

15 Valdivieso D (1964) La fauna quiróptera del Departamento de Cundinamarca, Colombia. Rev Biol Trop 12(1):19–45.

16 Morales-Alarcón A, Osorno-M E, Bernal CC, Llevas AP (1968) Aislamiento de virus rábico de murciélagos en Colombia, S. A.Caldasia 10: 167–72.

17 Jones Jr JK, Hood CS (1993) Synopsis of south American bats of the family Emballonuridae. Occas Pap Tex Tech Univ Mus 155:1–32

18 Alberico MS (1987) Notes on distribution of some bats from southwestern Colombia. In: Patterson BD, Timm RM, editos. Studies in Neotropical mammalogy, essays in honor of Philip Hershkovitz, Fieldiana Zool., 39: 133–36.

19 Castaño JH, Muñoz-Saba Y, Botero JE, Vélez JH (2003) Mamíferos del departamento de Caldas, Colombia. Biota Colomb. 4:247–59

20 Handley CO (1976) Mammals of the Smithsonian Venezuelan Project. Brigham Young Univ. Sci. Bull., biol. ser., 20(5):1–89.

21 Husson AM (1978) The mammals of Suriname.Zoölogische Monographieën van het Rijksmuseum van Natuurlijke Historie No. 2. Leiden: E. J. Brill, 160 p.
